# Circulating tumor DNA (ctDNA) detection is associated with shorter progression-free survival in advanced melanoma patients

**DOI:** 10.1038/s41598-020-75792-1

**Published:** 2020-10-29

**Authors:** Gabriella Taques Marczynski, Ana Carolina Laus, Mariana Bisarro dos Reis, Rui Manuel Reis, Vinicius de Lima Vazquez

**Affiliations:** 1 Molecular Oncology Research Center, Barretos Cancer Hospital, 1331, Antenor Duarte Villela St, Barretos, SP 14784-400 Brazil; 2 Life and Health Sciences Research Institute (ICVS), School of Medicine, University of Minho, Braga, Portugal; 3 ICVS/3B’s – PT Government Associate Laboratory, Braga, Guimarães, Portugal; 4 Surgery Department of Melanoma, Sarcoma and Mesenchymal Tumors, Barretos Cancer Hospital, Barretos, Brazil; 5 Barretos School of Health Sciences Dr. Paulo Prata - FACISB, Barretos, Brazil

**Keywords:** Tumour biomarkers, Melanoma, Prognostic markers

## Abstract

*BRAF*, *NRAS* and *TERT* mutations occur in more than 2/3 of melanomas. Its detection in patient’s blood, as circulating tumor DNA (ctDNA), represents a possibility for identification and monitoring of metastatic disease. We proposed to standardize a liquid biopsy platform to identify hotspot mutations in *BRAF*, *NRAS* and *TERT* in plasma samples from advanced melanoma patients and investigate whether it was associated to clinical outcome. Firstly, we performed digital polymerase chain reaction using tumor cell lines for validation and determination of limit of detection (LOD) of each assay and screened plasma samples from healthy individuals to determine the limit of blank (LOB). Then, we selected 19 stage III and IV patients and determined the somatic mutations status in tumor tissue and track them in patients’ plasma. We established a specific and sensitive methodology with a LOD ranging from 0.13 to 0.37%, and LOB ranging from of 0 to 5.201 copies/reaction. Somatic mutations occurred in 17/19 (89%) patients, of whom seven (41%) had ctDNA detectable their paired plasma. ctDNA detection was associated with shorter progression free survival (*p* = 0.01). In conclusion, our data support the use of ctDNA as prognosis biomarker, suggesting that patients with detectable levels have an unfavorable outcome.

## Introduction

Melanoma is the most aggressive type of skin cancer due to the high occurrence of metastases and resistance to conventional chemotherapy^1^. Moreover, melanoma rates have been rising for the last decades in predominantly fair-skinned populations and despite the favorable prognosis in early stages, local advanced or metastatic disease are still life-threatening conditions^2^. Currently, with the use of *BRAF/MEK* inhibitors and immunotherapy, the survival rates for metastatic melanoma have increased substantially. However, only a few patients produce durable responses, while the remainders develop resistance during treatment and experience disease progression at some point^3–6^.

The actual staging system from the American Joint Committee on Cancer (AJCC) includes prognostic and predictive biomarkers for melanoma^7^. However, AJCC only considers anatomical and histological features, which implicates that treatment decision and monitoring decisions are based solely on clinical and imaging findings, since there are no reliable blood-based biomarkers applicable for routine clinical use^8,9^. To rapidly identify metastatic progression and appropriately select patients for more aggressive therapy and avoid unnecessary treatments, there is a need for more tumor biomarkers.

In this context, a candidate biomarker that has been intensively evaluated is the circulating tumor DNA (ctDNA), which makes up part of cell-free DNA (cfDNA) from plasma^10,11^. The cfDNA comprises short fragments of DNA (160–180 base pairs), originated from cell death processes including necrosis and apoptosis and also cellular secretions^12^. The cfDNA concentration varies from 10 to 15 ng per milliliter of blood in healthy individuals, however it increases in certain conditions such as inflammation, exercise, tissue injury and in patients with cancer^13^. The ctDNA is the term for the fraction of cfDNA derived from tumor and therefore carries genetic alterations from the tumor. By the knowledge of tumor specific alterations, it is possible not only to identify molecular changes of the tumor but also to monitoring tumor burden in plasma. Recently, the detection of ctDNA alterations in melanoma patients has gain attention, due to its practical advantages: it is minimally invasive, allows serial collection, and is less prone to limitations such as quality of the material and tumor heterogeneity than traditional tissue biopsies^14–16^. Some relevant findings of this method include the association of ctDNA levels to tumor burden in metastatic melanoma patients and the ability of baseline values to predict outcomes^11,17,18^. The ctDNA variability during melanoma treatment is another possible tool for early assessment of therapy response or resistance^18–23^.

The most frequent and explored alterations are *BRAF* and *NRAS* mutations, present in 40–60% and 28% of patients, respectively^24,25^. In addition, a high frequency of mutations in the promoter region of *TERT* gene has been found in melanoma patients with a frequency ranging from 50 to 80% in several studies^24,26,27^, including Brazilian populations^28^. The most frequently described mutations are C228T and C250T, which are mutually exclusive^24^, but can co-occur with *BRAF* or *NRAS* mutations and are associated with lower survival^28–30^.

Therefore, we hypothesize that determining these driver mutations in tumor tissue and tracking them in plasma, it could contribute to identify melanoma patients at higher risk for progression. The aims of this study were to standardize a highly sensitive methodology of digital PCR (dPCR) to accurately identify hotspot mutations in *BRAF, NRAS,* and *TERT* in plasma and interrogate its feasibility as a liquid biopsy tool for melanoma patients.

## Results

### Establishment of optimal conditions of digital PCR

**Figure 1:**
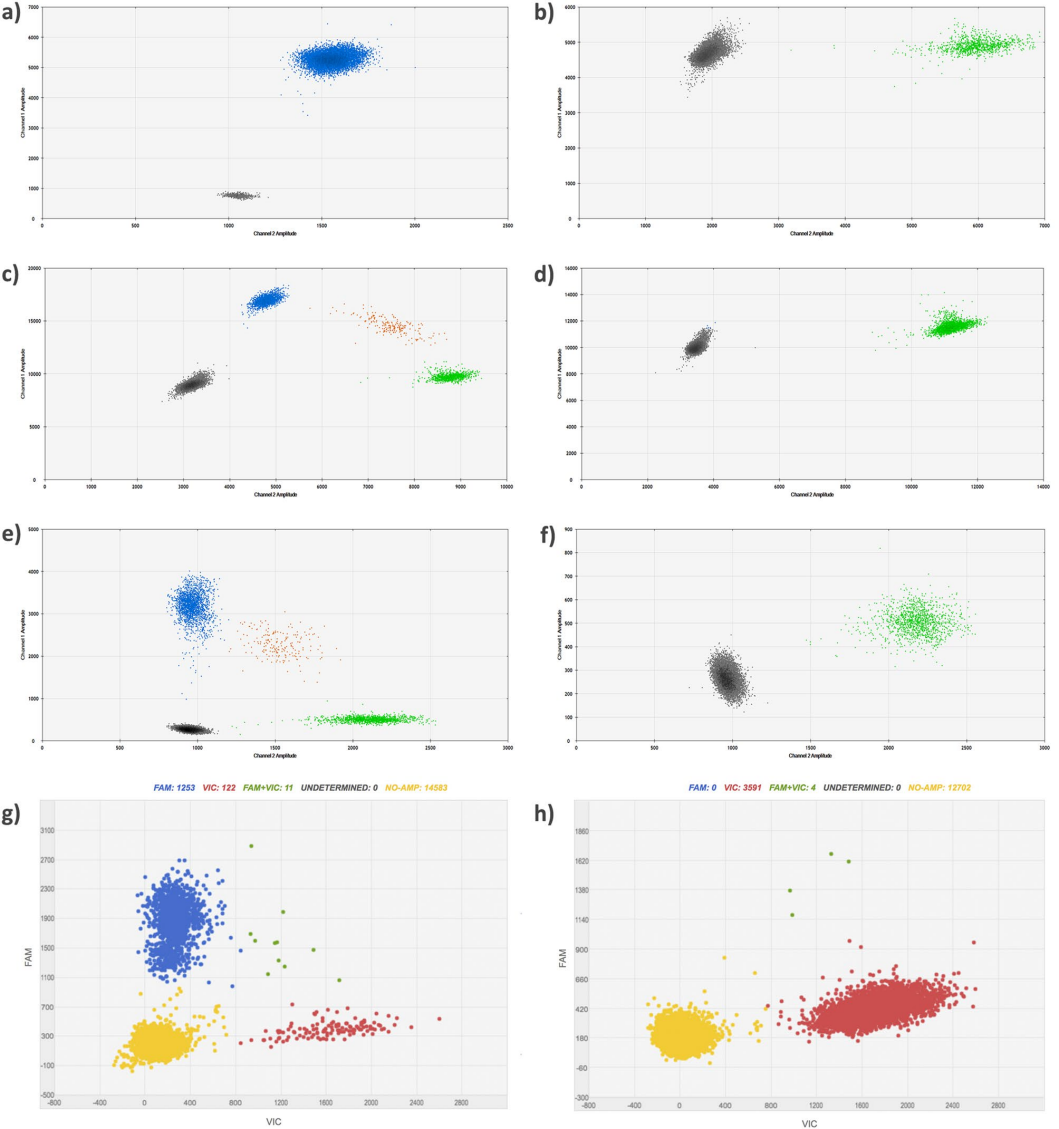
Scatter plots of *BRAF* (**a**, **b**), *NRAS* (**c**, **d**) and *TERT* (**e**, **h**) assays. In all plots, the y-axis represents mutant channel and the x-axis represents the wild type channel. (**a**) *BRAF* + UACC62 cell line. (**b**) *BRAF*- SKMEL103 cell line. (**c**) *NRAS* + SKMEL 103 cell line. (**d**) *NRAS*- UACC62 cell line. (**e**) *TERT* C250T + SIHA cell line. (**f**) *TERT* C250T- HS587T cell line. (**g**) *TERT* C228T + HS587T cell line on QuantStudio3D platform. (**h**) *TERT* C228T- SIHA cell line, presenting a small positive double population in the upper right corner (green), which in this case was considered false positive.

For each *BRAF*, *NRAS*, *TERT* C250T and *TERT* C228T mutation detection assay, we performed temperature gradients to identify optimal annealing extension temperatures and times. Both *BRAF* and *NRAS* assays demonstrated better clustering between populations at 53 °C of annealing temperature, as shown in Fig. 1a–d. For *BRAF* mutation assay, the positive control (UACC62–*BRAF* V600E) showed two populations, the double negative droplets and the FAM positive (mutant) droplets, since the cell line harbor the V600E mutation in homozygosis (Fig. 1a). The *BRAF* negative control (SKMEL103 melanoma cell line), besides empty droplets, presented a population only on the y-axis, being correctly classified as wild type (Fig. 1b). For *NRAS* assay, the Q61R mutation positive control (SKMEL103) exhibited the mutation in heterozygosis, and therefore showed in the two dimensional plot representation of the four populations: the mutated homozygous droplets in blue, the droplets with the two alleles in orange, the wild type only droplets in green, and the double negative droplets in gray (Fig. 1c). As expected, the wild type control for *NRAS* (UACC62), presented double negative, and wild type droplets (Fig. 1d).

Due to the design characteristics of the oligonucleotides and amplification target region, both *TERT* assays required different cycling conditions. For *TERT* mutation the optimal conditions were under 54 cycles with annealing temperature of 59 °C (Fig. 1e). The positive control, heterozygous for *TERT* C250T mutation (SIHA cervical cell line), presented the four populations while negative control (HS587T breast cell line) presented double negative and the wild type droplets population (Fig. 1e, f, respectively). For the *TERT* C228T assay, despite extensive optimization attempts, the assay did not perform well in QX200 Droplet Digital PCR (Bio-Rad Laboratories, USA), presenting probe cross-reactivity. Instead, we benefit from testing *TERT* C228T in Quant Studio 3D (ThermoFisher Scientific, USA) equipment, using 54 cycles with annealing temperature of 55 °C (according to the manufacturer's recommendation). The positive control for *TERT* C228T (HS587T) had a similar distribution to the other heterozygous cell line previously demonstrated, with four populations (Fig. 1g). The wild type control (SIHA) presented some false-positive reactions (Fig. 1h).

### Determination of limit of blank (LOB), limit of detection (LOD) and linearity evaluation

The limits of blank (LOBs) for each assay were calculated as described and results are summarized in Supplementary Table S1. For *BRAF* assay, since no mutant copies were observed in any control, the LOB was 0 copies/reaction. For *NRAS* assay, we found a LOB of 2.13 copies/reaction. Both *TERT* assays had a higher level of false-positive rates with LOBs of 3.995 and 5.201 copies/reaction for C228T and C250T, respectively (Supplementary Table S1).

**Figure 2:**
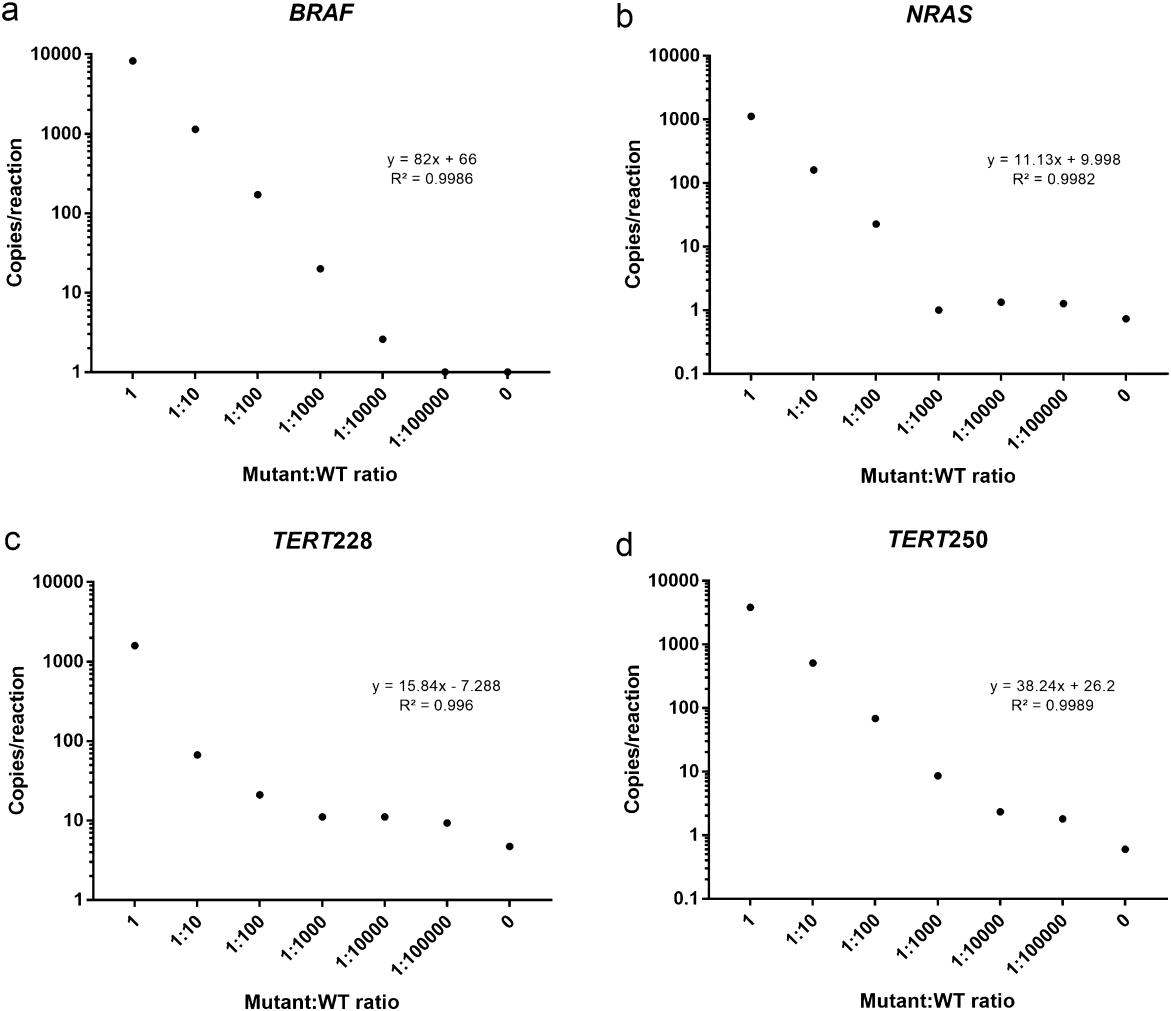
Serial dilution of mutant DNA from cell lines in a wild type background DNA. The number mutant DNA copies determined by dPCR were plotted against the corresponding dilutions. (**a**) Dilution of DNA from cell line UACC62 harboring *BRAF* V600E mutation. (**b**) Dilution of DNA from cell line SKMEL 103 harboring *NRAS* Q61R mutation. (**c**) Dilution of DNA from cell line HS587T harboring *TERT* C228T mutation. (**d**) Dilution of DNA from cell line SIHA harboring *TERT* C250T mutation.

The next step was to evaluate the linearity of assays and determine the analytical sensitivity of each assay. Using replicates of six dilutions of mutant in wild type DNA background, (Fig. 2). A high linearity between the expected and the measured fraction of mutant DNA was observed with a R^2^ = 0.9986 for *BRAF* (Fig. 2a), R^2^ = 0.9982 for *NRAS* (Fig. 2b), R^2^ = 0.996 for *TERT* C228T (Fig. 2c) and R^2^ = 0.9989 for *TERT* C250T (Fig. 2d).

The lowest concentration sample dilution was used to determine the LOD of assays, calculated as described by Armbruster and Pry^31^, varied from 3.71 to 9.76 copies/reaction with fractional abundance of 0.13% to 0.37% (Supplementary Table S1).

### Patient demographics features and detection of baseline ctDNA

**Figure 3:**
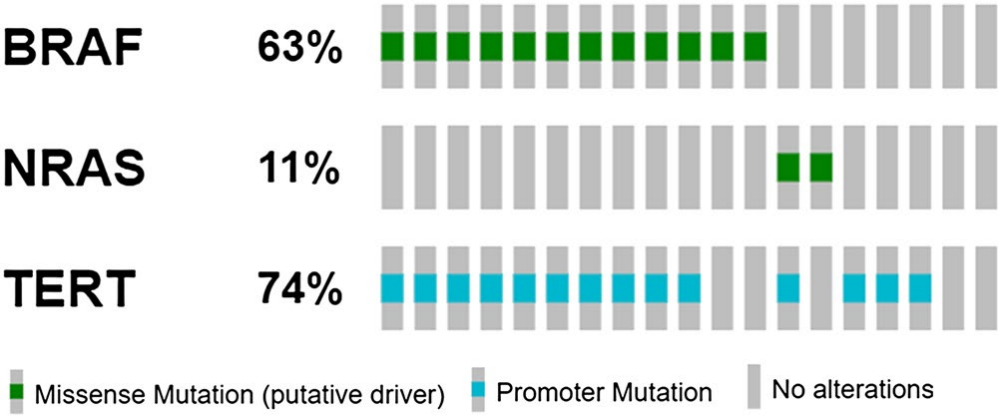
Somatic mutation profile determined on FFPE samples. Patients are classified according to presence of mutations in *BRAF, NRAS, TERT* or TRIPLE negative/wild type.

**Figure 4:**
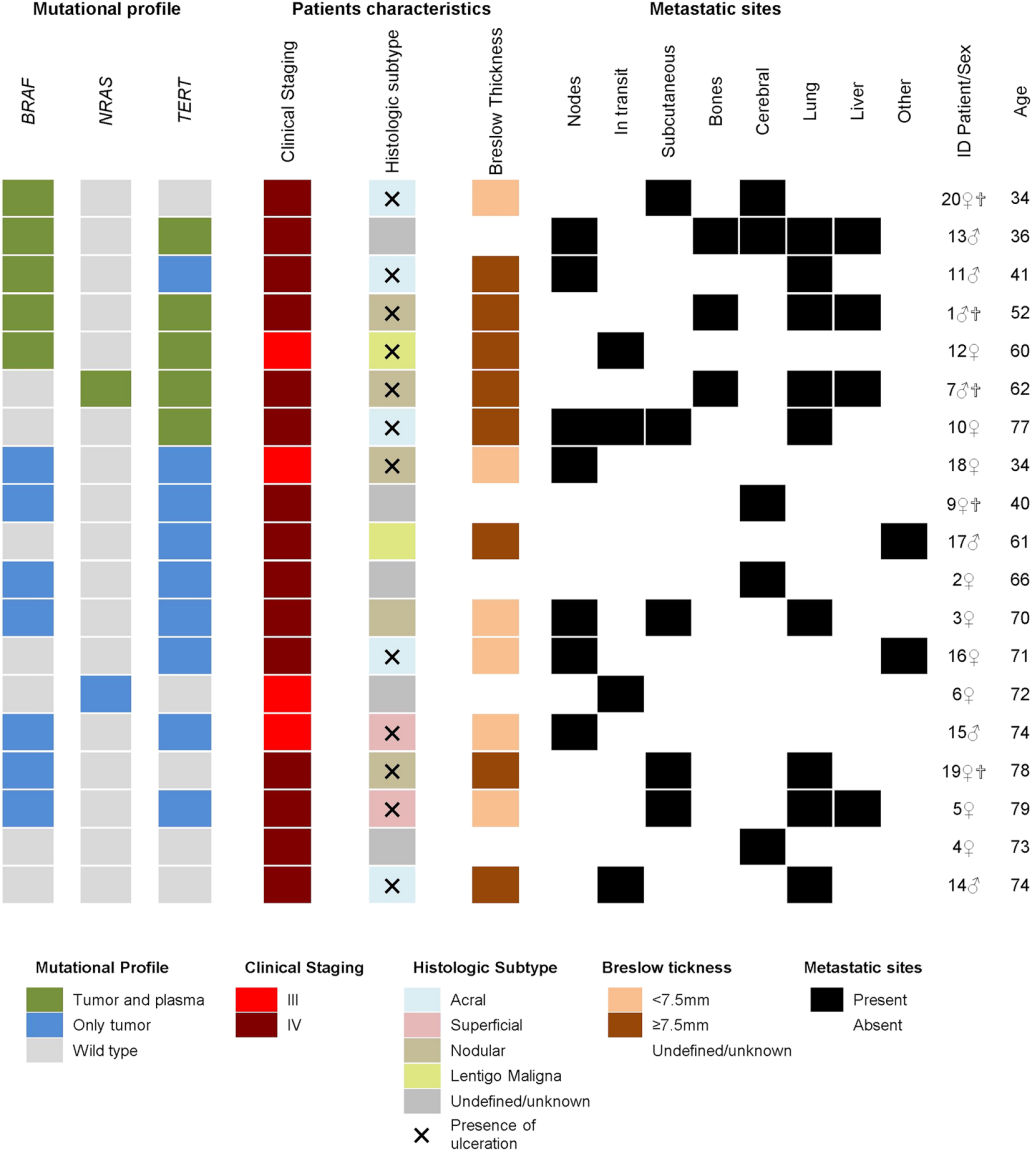
Overview of patient’s mutational profile, characteristics, and site of metastasis. Patients are ordered according to ctDNA detection.

*BRAF, NRAS,* and *TERT* somatic mutations were evaluated in patient’s tumor tissue as part of clinical routine (Fig. 3). From 19 patients analyzed, 17 (89%) had at least one somatic mutation. Twelve patients had *BRAF* mutations (63.2%—10 patients: V600E, 2 patients—V600K), while two patients (10.5%) presented *NRAS* mutation (1 patient—Q61H; 1 patient—Q61R) and 14 patients had *TERT* alterations (74%) (7 patients—C228T; 7 patients—C250T). Most patients (n = 11) presented more than one gene altered, and as expected *BRAF* and *NRAS* were mutually exclusive. Two patients were triple negative for selected genes (IDs 4 and 14) (Fig. 4).

Clinical information of the nineteen patients are summarized in Fig. 4. Overall, 12 (63.2%) patients were female and 7 (36.8%) were male, the ages ranged from 34 to 79 years, with a mean of 61 years, 15 (79%) had distant metastases (stage IV) and 4 (21%) presented only lymph node involvement (stage III). The most frequent histological subtypes were acral and nodular melanoma (5 patients each). Two patients (IDs 4 and 9) had only brain metastasis with unknown primary site and 3 patients had no information on the primary lesion, being classified as an unknown histological subtype. Regarding the Breslow thickness of the primary lesion, both the mean and the median thickness of the group were around 7.5 mm, so that the patients were grouped according to this value, above and below (Fig. 4). Finally, 5 patients died and 14 are still being followed up.

ctDNA could be detected in seven (41%) of the 17 patients that had at least one mutation identified in the tumor tissue. Quantification of mutant targets (copies/reaction) for each assay is described in Supplementary Table 1 (Table S2). Analyzing by gene, *BRAF* mutations were detected in plasma from in 5 of 12 cases (41.6%), in 1 of 2 cases of *NRAS* positive (50%), and in 5 of 14 *TERT* positive mutations (35.7%). From 12 patients that carried two genes mutated on tumor tissue, both alterations were detected in plasma in 4 cases (33.3%), in 7 patients, neither mutation was detected in ctDNA, and 1 patient had only one of the mutations observed (8.3%). Patients without mutation in the FFPE tissue did not exhibited mutation in the plasma (Fig. 4).

### Association of ctDNA and patients’s prognostic factors

We grouped the 17 patients according to ctDNA detection in plasma: detectable (n = 7) and undetectable (n = 10). The association between the clinical features of the 17 patients and ctDNA detection is shown in Table 1. Of the patients with detectable ctDNA, 5 (71%) were younger patients (< 61 years). It was observed a tendency between Breslow thicknesses of primary lesions greater than 7.5 mm with detectable ctDNA, yet it was not significant. Also, a higher frequency of detectable ctDNA was observed in patients with more than one metastasis site. Individuals with isolated central nervous system (CNS) metastases had no detectable ctDNA (IDs 2 and 9).

**Table 1:** Association of ctDNA with melanoma patient’s clinical features (n = 17).

Variables	Detectable	Undetectable	*p* value
**Sex**^**a**^			
Female	3 (27%)	8 (73%)	0.162
Male	4 (67%)	2 (33%)	
**Age**^**a,b**^			
< 61 years	5 (71%)	2 (29%)	0.058
≥ 61 years	2 (20%)	8 (80%)	
**Clinical stage**^**a**^			
III	1 (25%)	3 (75%)	0.603
IV	6 (46%)	7 (54%)	
**Histological subtype**^**a**^			
Acral	3 (75%)	1 (25%)	0.435
Superficial	0 (0%)	2 (100%)	
Nodular	2 (40%)	3 (60%)	
Lentigo Maligna	1 (50%)	1 (50%)	
Undefined/unknown	1 (25%)	3 (75%)	
**Breslow thickness**^**a.c**^			
< 7.5 mm	1 (17%)	5 (83%)	0.103
≥ 7.5 mm	5 (71%)	2 (29%)	
Undefined/unknown	1 (25%)	3 (75%)	
**Ulceration**^**a**^			
Absent	0 (0%)	2 (100%)	0.267
Present	6 (55%)	5 (45%)	
Undefined/unknown	1 (25%)	3 (75%)	
**Site of distant metastasis**^**a**^			
M0	1 (25%)	3 (75%)	0.723
M1a	0 (0%)	1 (100%)	
M1b	1 (33%)	2 (67%)	
M1c	3 (60%)	2 (40%)	
M1d	2 (50%)	2 (50%)	
**Number of distant metastasis**^**a,d**^			
1 Sites of metastasis	1 (20%)	4 (80%)	0.266
> 2 Sites of metastasis	5 (63%)	3 (37%)	
**Number of somatic mutations**^**a**^			
One	2 (29%)	5 (71%)	> 0.999
Two	5 (50%)	5 (50%)	
**Mutational status**^**a**^			
*BRAF*	1 (50%)	1 (50%)	0.685
*NRAS*	0 (0%)	1 (100%)	
*TERT*	1 (33%)	2 (67%)	
*BRAF* + *TERT*	4 (40%)	6 (60%)	
*NRAS* + *TERT*	1 (100%)	0 (0%)	

^a^Analysis by Fisher’s exact test expressed as absolute (n) and relative (%) frequencies;

^b^Categories defined from mean;

^c^Categories defined from median;

^d^Only stage IV patients.

### Association of ctDNA and patient outcome

**Figure 5:**
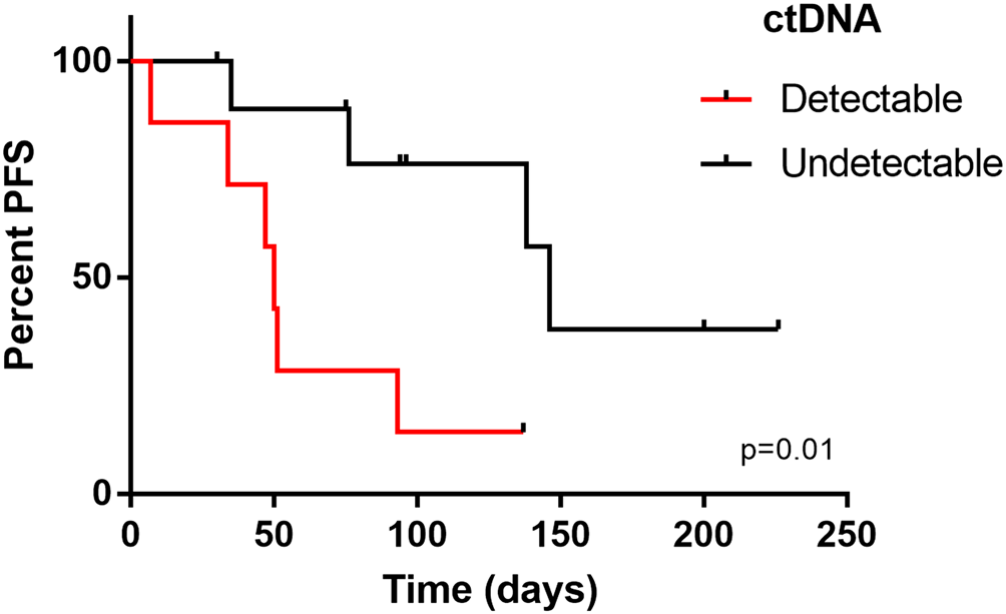
Kaplan–Meier curve Association between detectable ctDNA and progression free survival (median PFS, 50 days for detectable ctDNA and 146 days for undetectable ctDNA, *p* = 0.014).

The median follow-up of patients was 130 days, ranging from 7 to 253. Patients who had detectable levels of ctDNA had a shorter progression-free survival when compared to patients with undetectable mutation (*p* = 0.01) (Fig. 5). The median time in days for progression in patients with circulating tumor DNA was 50 days, and in patients without circulating tumor DNA it was 146 days. Patients with detectable ctDNA had significantly increased risk of progression compared with those with undetectable ctDNA (HR: 6.38; 95% CI 1.26–32.1; *p* = 0.025).

## Discussion

The emergence of target therapy and immunotherapy has contributed significantly to the survival of patients with metastatic melanoma^4^. However, melanoma lacks prognostic and predictive biomarkers, which implicates on the challenges in select patients with a poorer prognosis that will benefit from a more aggressive therapy and for early identification of disease progression.

Here, we validated a dPCR approach that detects the most frequent melanoma alterations in cfDNA in a real-world context. We demonstrated that these specific mutations are present in tumor tissue of 89% of our patients, and taking together, mutations could be detected in plasma from seven of seventeen patients (41%). Briefly, ctDNA was detected in 5 of 12 cases *BRAF* positive (41.6%), in 1 of 2 cases *NRAS* positive (50%), and in 5 of 14 *TERT* positive mutations (35.7%).

Liquid biopsy appears as a minimally invasive model, low-cost, sensitive, reproducible and with a wide variety of applications in personalized medicine for melanoma through analysis of circulating tumor DNA^11,25,32^. In attempt to implement liquid biopsy in a clinical setting, our group sought to standardize a platform to evaluate hotspot mutations in melanoma patients. In addition to *BRAF* and *NRAS* alterations, which are the most explored genes in most studies^33^, we included the *TERT* gene, since mutations in its promoter region have been described by its high frequency in melanoma patients^27–29^. The dPCR methodology was chosen due to its high sensitivity and accuracy, especially in the detection of rare alleles at low frequencies, that best fits in this context^34,35^. The major disadvantage of dPCR is that, generally, only one target per test can be evaluated in dPCR, which implicate higher cost and time consuming, when analyzing a large number of genes. Even that multiplex assays have been developed recently, there is a limitation in number of targets to be included, due to the necessity that PCR conditions must be the same for all targets. Besides that, the majority of dPCR systems have only two channels of fluorescence detection (FAM and VIC/HEX), which can limit the design of assays with many targets.

Other methods of ctDNA detection can be used, as Next-generation sequencing (NGS). NGS can provide not only the ability to detect genome-wide DNA variation but also the use of multiple targeted panels^36^. The use of these panels has lower cost compared to whole-genome-sequencing (WGS) and whole-exome sequencing (WES) and also offers the advantage of reducing the DNA input and still achieving a high sensitivity. However, the detection of rare alleles at a low frequency requires a very high coverage (> 10.000×), which increases sequencing cost^37^. In addition, there is an important limitation in the analysis of *TERT* promoter mutations in NGS panels. Despite validated for FFPE NGS panels^38^, *TERT* promoter mutations have not performed well for cfDNA in NGS custom panels^39,40^ due to its high GC content, and so, it is mostly described in droplet digital PCR approaches^41,42^. Furthermore, *BRAF* and *NRAS* mutations are mutually exclusive, as well as *TERT* C250T and C228T, and then, a maximum of two mutations will be tested in plasma for each melanoma patients, making feasible the dPCR approach proposed in this study.

We detected plasma ctDNA in seven of seventeen patients who had at least one somatic mutation in tumor tissue. This is in agreement with three other similar studies using ddPCR, which indicated that ctDNA detection rates are associated with tumor burden^18,23,43^. McEvoy *et al*^18^ demonstrated a detection rate of 71.8% in a cohort of 32 stage IV patients. In keeping with the relationship to burden, detection rates fell to 12% in lower stage patients (EC II and III)^23^. The same group showed a detection rate of 53% at baseline in stage IV patients that undergone treatment with PD1 inhibitors^22^. Data from Braune and colleagues, showed an even higher detection rate of 87.1% in a group of 62 patients that comprised 50 stage IV and 12 stage III melanoma patients^43^. The fact that our group comprised both stage III and IV indicates that our detection rate was similar to other studies examining these stages. However, the study from McEvoy, demonstrated that ctDNA detection not only depends on the clinical stage, but also on the metabolic tumor burden (MTB) and metabolic tumor volume (MTV) accessed by 18F-labeled fluorodeoxyglucose positron emission tomography/computed tomography, as ctDNA was undetectable in all patients with an MTB value of ≤ 10^18^.

Another important aspect associated to ctDNA detection is the volume of plasma processed and the input of DNA in dPCR reaction, as the screening of small amounts of sample may not always represent the true mutant content, mainly when they are present in a few copies in our sample. The high detection rate from Braune et al. might be associated to the higher input screened. Despite using 2 ml of plasma they tested samples in 4 replicates^43^. Corless and colleagues screened 12 replicates from plasma samples for *TERT* evaluation in ddPCR^42^. Some other studies isolated DNA from a larger volume of plasma, up to 5mL^41,44^. We presume the most important limitation of our assay was the fact that it was possible to used only 2 mL of plasma and 10 ng per reaction, which generated a higher LOB and, consequently LOD. Higher cut-offs implicates in lower sensitivity. It can have affected the accuracy of our experiment in diagnostic sensitivity, but it demonstrated to be important for determining patient prognosis.

*TERT* mutation occurs in up to 80% of cutaneous melanomas, which makes this gene extremely important to be part of the panel proposed^26–29^. However, it is established that the high GC content (approximately 80%) in the promoter region of the gene imposes difficulties in the design of an efficient and specific PCR assay to detect and quantify mutations in this region^42^. This was a technical challenge with *TERT* C228T mutation. The assay chosen in this study was designed by ThermoFisher Scientific company, but even after several approaches, it was not possible to standardize the assay in the Bio-Rad platform. However, the analysis of this mutation in QuantStudio 3D systems achieved reliable results, as demonstrated by our results both with cell lines and plasma samples.

Despite preliminary, our data suggest an association between ctDNA and worse prognosis (HR: 6.38; 95% CI 1.26–32.1; *p* = 0.025), as previously described^11,18,19,21–23,41,45–47^. From patients with detectable ctDNA, 85% of patients were stage IV, whilst the only stage III patient (ID 12) progressed in 50 days. It is in accordance to other studies that show that the disease burden influences the detection of ctDNA^17,18^. Also, as reported in literature, ctDNA was not detected in patients with exclusive central nervous system metastases, a fact that could be explained by the presence of the blood–brain barrier, which prevents the detection of circulating DNA^21^.

## Conclusion

Our study demonstrates the impact of ctDNA detection in patient prognosis, in which patients with detectable levels have a worse outcome. Further studies should be done to extend and validate these findings. Moreover, a longitudinal study with several samplings will also allow evaluating the variations in ctDNA levels in patients during the treatment to determine the clinical benefit of the therapies.

## Materials and methods

### Patient recruitment and study design

We selected naïve-treated melanoma patients with advanced clinical stage III and IV, admitted at Melanoma and Sarcoma Department of Barretos Cancer Hospital from March to November of 2018, or had a follow up appointment during this period. Formalin-fixed paraffin-embedded (FFPE) and peripheral blood samples from patients were collected prospectively at baseline. The present study was approved by the Human Research Ethics Committee of the Barretos Cancer Hospital (1465/2017) and all participants gave their informed written consent. All methods were carried out in accordance with relevant guidelines and regulations.

### Identification of somatic mutation

Somatic mutation of hotspot regions of *BRAF*, *NRAS*, and *TERT* genes was determined on formalin-fixed paraffin-embedded (FFPE) tissue available from Pathology Department. Detection of mutation in *BRAF* and *NRAS* were performed using the TruSight Tumor 15 panel (Illumina, USA) of next generation sequencing (NGS) as standardized in our institution^48^. For *TERT*, mutation analysis was performed by Sanger Sequencing^28^, or dPCR methodology in those cases that DNA quality did not allow a reliable result from sequencing.

### Plasma processing and cfDNA isolation

Between 5 and 10 mL of whole blood were collected in a tube containing EDTA and processed within four hours after collection for plasma separation. To separate plasma from buffy coat, centrifugation was done at 3500 rpm for 10 min and then plasma was stored in 1 mL aliquots in − 80 °C freezer until DNA isolation. cfDNA was isolated from 2 mL of the plasma with the commercial QIAamp Circulating Nucleic Acid Kit (Qiagen, Germany), according to the manufacturer’s protocol and eluted in 35 μL of ultrapure water, quantified using the dsDNA High Sensitivity Assay Kit and Qubit 2.0 Fluorometer (ThermoFisher Scientific, USA) and stored at − 20 °C.

### Assays design and Digital PCR platforms

All assays are based on the principle of genotyping, with primer pair delimiting the region of interest and there are two fluorescence/color-labeled probes, one detecting the mutated allele (FAM) and the other one, the wild type allele (HEX/VIC). For *BRAF* and *NRAS*, commercial Bio-Rad assays were used (ddPCR *BRAF* V600 Screening Kit 12001037, capable of detecting mutations in V600E/K/R and ddPCR *NRAS* Q61 Screening Kit 12001006, capable of detecting mutations in Q61K/L/R/H 183A > T/183A > C). For mutation evaluations in *TERT* promoter region, TaqMan (ThermoFisher Scientific, USA) assays were used for each mutation, including Hs000000092 (ThermoFisher Scientific, USA) commercial assay for *TERT* C228T mutation and Hs000000093 (ThermoFisher Scientific, USA) commercial assay for C250T mutation.

*BRAF*, *NRAS* and *TERT* C250T mutations were evaluated in QX200 Droplet Digital PCR (ddPCR) system (Bio-Rad Laboratories, USA). For ddPCR reaction mixture, 1× ddPCR Supermix for Probes (No dUTP), 1× of specific assay and a volume from 1 to 9 μL of DNA were mixed, depending on the concentration of the sample. The final volume of 20 μL of the reaction, together with 70 μL of the Droplet Generation Oil for Probes, was submitted to the QX200 Droplet Generator. PCR reactions were performed in C1000 Touch Thermal Cycler with 96-Deep Well Reaction, droplets were read in the QX200 Droplet Reader and analyzed using Quanta Soft software (Bio-Rad Laboratories, USA). The *TERT* C228T assay was performed on the Quant Studio 3D platform (ThermoFisher Scientific, USA), after a series of optimization that failure in Bio-Rad platform. For the reaction, 1× Quant Studio 3D Digital PCR Master Mix v2, 1× of the specific assay and 1–6.6 μL of DNA were mixed according to the concentration of the sample. The final volume of 14.5 μL was applied directly to the Quant Studio 3D Digital PCR 20 K Chip v2, and PCR reaction occurred in the Pro Flex thermal cycler using the chip-specific adapter. The chip was inserted into Quant Studio 3D for fluorescence reading and then analyzed by Analysis Suite software (ThermoFisher Scientific, USA).

### Validation, optimization, and determination of linearity of DNA amplification

To determine assay’s ability to differentiate mutation positive from mutation negative samples, we tested commercial tumor cell lines whose mutational status are known (Table 2). We evaluated optimal amplification conditions for each assay by testing 20 ng of DNA to a range of temperatures above and below the melting temperature in order to choose the one who provides the optimal cluster separation. Next, a serial dilution containing a mutant ratio of 1:10, 1:100, 1:1000, 1:10,000 and 1:100,000 in a background of wild type DNA (n = three independent replicates) were performed to evaluate the performance and analytical sensitivity of the assays. For this experiment, we tested 10 ng, which is the expected yield for testing samples (cfDNA). Sensitivity was defined as the lowest concentration likely to be reliably distinguished from blank samples.

**Table 2:** Description of tumor cell lines used for validation of commercial assays.

	Source	Mutation
UACC62^a^	Melanoma	*BRAF* V600E (c.1799 T > A)
SKMEL 103^a^	Melanoma	*NRAS* Q61R (p.Gln61Arg)
SIHA^b^	Cervical squamous cell carcinoma	*TERT* -146G > T (C250T)
HS587T^b^	Breast carcinoma	*TERT* -124C > T (C228T)

^a^Cell lines kindly provided by Dr. Silvya Stuchi Maria Engler—University of Sao Paulo.

^b^Cell lines acquired from ATCC.

For the evaluation of assay linearity we compared the concentration in copies/reaction to the expected serial dilution with a linear regression.

### Determination of limit of blank (LOB) and limit of detection (LOD)

In order to determine the false positive rate for each assay and accurately determine the limit of detection, we analyzed cell-free DNA extracted from plasma donated by 20 healthy volunteers (blank samples). The LOB was calculated as described by Armbruster and Pry^31^, where: LOB = meanblank + 1.645*SDblank.

After establishing the LOB, we calculated the lowest analyte concentration likely to be reliably distinguished from the background (LOD) as follow: LOD = LOB + 1.645*(SD low concentration sample)^31^.

### Data analysis of patient samples and positive calls

PCR reactions for patient samples were performed using a maximum input of 10 ng of cfDNA. Each run included mutant positive controls, wild type controls, and non-template controls.

For quality analysis, the overall accepted events/reading should be above 10,000 according to manufacturer’s protocol. To determine positive droplets, the threshold was set examining the three controls wells using the 2D plot. Samples were classified as mutated when number of copies/reaction exceeded the LOB calculated for that assay.

The number of mutated DNA copies per reaction was used to calculate copies per mL of plasma using the following equation:

Copies/mL of plasma = C*EV/TV/PV. PV = Volume of plasma used for cfDNA extraction (mL) EV = Volume in which cfDNA was eluted (μL), TV = Volume of cfDNA added to the PCR reaction (μL), C = copies/reaction (data derived from QuantaSoft or Analysis Suite softwares).

### Statistical analysis

Association between patient detection of ctDNA (undetectable/detectable) and patient’s characteristics (gender, age, AJCC stage, histological classification, primary melanoma Breslow and ulceration, site of metastasis, as well as mutational status) were performed using a Fisher’s exact test. Progression free survival (PFS) was calculated as the time from the date of collection/inclusion to the date of the first disease progression (PD) clinically defined or censored with stable disease at the most recent visit. Median PFS was calculated using the Kaplan–Meier method and compared using the log-rank test. A Cox proportional hazards regression analysis was performed to examine association of ctDNA detection with PFS. Statistical analysis was performed using the IBM SPSS Statistics for Windows version 20.0.

All results were considered significant when *p* value was < 0.05. Clinical, demographic, histopathological, and treatment-related data, as well as disease progression or relapse data were prospectively collected using a questionnaire and medical records and stored in an electronic database (REDCap).

## Supplementary information

Supplementary Information
