# Supplementary material for: Circulating tumor DNA (ctDNA) detection is associated with shorter progression-free survival in advanced melanoma patients
Source: Sci Rep. 2020 Oct 29;10:18682. doi: 10.1038/s41598-020-75792-1 (PMC7596487; doi:10.1038/s41598-020-75792-1)
Supplement: Supplementary file 1 — Supplementary Information [file 41598_2020_75792_MOESM1_ESM.docx]

Circulating tumor DNA (ctDNA) detection is associated with shorter progression-free survival in advanced melanoma patients

Gabriella Taques Marczynski^1^, Ana Carolina Laus^1^, Mariana Bisarro dos Reis^1^, Rui Manuel Reis^1,2,3^, Vinicius de Lima Vazquez^1,4,5^*

Table S1: The limit of blank (LOB) calculated for each assay and the lowest concentration of mutant template that was detected in calculated dilutions (mut:wt ratio) were used to calculate the limit of detection (LOD). The LOB denotes false positivity or specificity of the assay, whereas LOD is the lowest analyte concentration likely to be reliably distinguished from the LOB and at which detection is feasible and approximates to the analytical sensitivity of the assay.

| Mutation assay | LOB (copies/reaction) | Mutant concentration (copies/reaction) in replicates of lowest concentration sample | LOD (copies/reaction) | Fractional abundance |
| --- | --- | --- | --- | --- |
| *BRAF* | 0 | 4; 0; 2.8 | 3.71 | 0.13% |
| *NRAS* | 2.213 | 18; 26; 24 | 9.06 | 0.37% |
| *TERT* 250 | 5.201 | 12.2; 5.2; 8.4 | 9.76 | 0.30% |
| *TERT* 228 | 3.995 | 11.107 | 3.995 | 0.23% |

Table S2: The number of mutated DNA copies per 20 μl reaction was extrapolated to calculate copies per mL using the following equation: copies/mL of plasma = C*EV/TV/PV. PV = Volume of plasma used for cfDNA extraction (ml) EV = Volume in which cfDNA was eluted (μl) TV = Volume of cfDNA added to the PCR reaction (μl) C = copies/reaction (data derived from QuantaSoft or Analysis suite softwares).

| PATIENT ID | ASSAY | CONCENTRATION (copies/mL) | Mutant allele frequency (MAF) |
| --- | --- | --- | --- |
| 1 | *BRAF* | 5034 | 50.3% |
| 11 | *BRAF* | 4 | 0.5% |
| 12 | *BRAF* | 30 | 1.2% |
| 13 | *BRAF* | 26704 | 57.9% |
| 20 | *BRAF* | 240 | 27.3% |
| 7 | *NRAS* | 76028 | 42% |
| 7 | *TERT 228* | 4492 | 3.91% |
| 10 | *TERT 228* | 21 | 0.757% |
| 1 | *TERT 228* | 373 | 5.33% |
| 12 | *TERT 228* | 21 | 0.73% |
| 13 | *TERT 228* | 224 | 12.2% |
